# The Effect of Arterial Curvature on Blood Flow in Arterio-Venous Fistulae: Realistic Geometries and Pulsatile Flow

**DOI:** 10.1007/s13239-017-0321-2

**Published:** 2017-07-26

**Authors:** L. Grechy, F. Iori, R. W. Corbett, W. Gedroyc, N. Duncan, C. G. Caro, P. E. Vincent

**Affiliations:** 1 Department of Aeronautics, Imperial College London, South Kensington, London, SW7 2AZ UK; 2 Imperial College Renal and Transplant Centre, Hammersmith Hospital, London, W12 0HS UK; 3 St Mary’s Hospital, Praed Street, London, W2 1NY UK; 4 Department of Bioengineering, Imperial College London, South Kensington, London, SW7 2AZ UK

**Keywords:** Haemodynamics, Anastomosis, Arterio-Venous Fistula, AVF, Blood flow CFD, Blood flow unsteadiness, Hypoxia

## Abstract

Arterio-Venous Fistulae (AVF) are regarded as the “gold standard” method of vascular access for patients with End-Stage Renal Disease (ESRD) who require haemodialysis. However, up to 60% of AVF do not mature, and hence fail, as a result of Intimal Hyperplasia (IH). Unphysiological flow and oxygen transport patterns, associated with the unnatural and often complex geometries of AVF, are believed to be implicated in the development of IH. Previous studies have investigated the effect of arterial curvature on blood flow in AVF using idealized planar AVF configurations and non-pulsatile inflow conditions. The present study takes an important step forwards by extending this work to more realistic non-planar brachiocephalic AVF configurations with pulsatile inflow conditions. Results show that forming an AVF by connecting a vein onto the outer curvature of an arterial bend does not, necessarily, suppress unsteady flow in the artery. This finding is converse to results from a previous more idealized study. However, results also show that forming an AVF by connecting a vein onto the inner curvature of an arterial bend can suppress exposure to regions of low wall shear stress and hypoxia in the artery. This finding is in agreement with results from a previous more idealized study. Finally, results show that forming an AVF by connecting a vein onto the inner curvature of an arterial bend can significantly reduce exposure to high WSS in the vein. The results are important, as they demonstrate that in realistic scenarios arterial curvature can be leveraged to reduce exposure to excessively low/high levels of WSS and regions of hypoxia in AVF. This may in turn reduce rates of IH and hence AVF failure.

## Introduction

End-Stage Renal Disease (ESRD) is characterized by an irreversible loss of kidney function, and is treated predominantly with haemodialysis or renal transplantation.^25^,^27^,^61^ Efficient haemodialysis is dependent upon high-quality vascular access, allowing the rapid removal of blood (at up to 450 mL min^−1^), which passes through a dialyser removing metabolic waste and water, before being returned to the body. The preferred method of vascular access^60^ is *via* an established Arterio-Venous Fistula (AVF) created surgically by connecting a patient’s own artery and vein, usually in the arm. The connection or “anastomosis” can take a number of configurations. However, an end-to-side arrangement, where the end of the vein is connected to the side of the artery, has emerged as the clinically preferred configuration.^36^

Following creation of an AVF, there is a rapid increase in blood flow within the artery and vein. In an ideal situation the venous lumen proceeds to enlarge, along with concurrent remodeling of the vessel wall.^20^ After a period of six to twelve weeks the vein should have “matured” such that it can support insertion of two large gauge needles several times a week - providing access for high quality haemodialysis over many years. Unfortunately, up to 60% of AVF do not mature as anticipated^1^ resulting in unacceptable patient morbidity and a significant burden on healthcare expenditure. Failure occurs predominantly due to the development of Intimal Hyperplasia (IH). Material obtained from immature dialysis access reveals a hyperplastic lesion arising from the intima that progressively occludes the lumen of the vessels, resulting in slow flow, and ultimately thrombosis and irreversible loss of the AVF.^2^,^50^ Specifically, IH is found in the venous section of the AVF close to the anastomosis^2^,^8^,^56^ and more distally,^8^,^56^ as well as in the arterial section of the AVF close to the anastomosis^6^–^8^,^56^ and more proximally.^6^,^8^

Whilst the exact mechanisms underlying development of IH are unknown, there is considerable evidence to suggest that the inherently unphysiological flow patterns within AVF play an important role. In particular regions of highly oscillatory flow, abnormally low/high Wall Shear Stress (WSS), and low Lumen-to-Wall Normal Oxygen Flux (LWNOF)—leading to wall hypoxia, have all been implicated in the initiation of IH.^10^,^13^,^14^,^16^,^19^,^22^,^23^,^26^,^30^,^38^,^39^,^45^,^46^,^49^,^58^,^59^ In 2015 Iori *et al.* 32 investigated the effect of planar arterial curvature on blood flow and oxygen transport in AVF using an idealized model. Specifically, idealized straight venous sections were connected to either the inside or outside of a curved planar arterial bend, with non-pulsatile inflow conditions. It was found that inner configurations suppressed potentially pathological low WSS and low LWNOF but, conversely, outer configurations suppressed potentially pathological high-frequency flow unsteadiness. The present study extends this work to more realistic non-planar AVF configurations with pulsatile inflow conditions.

## Methods

### Geometries

#### Native Vessels

**Figure 1:**
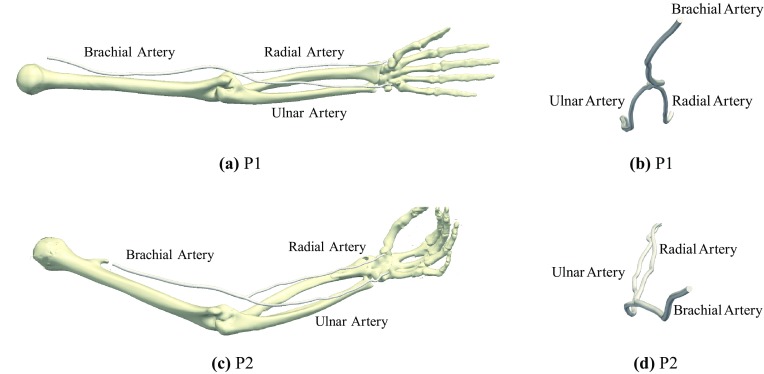
Longitudinal (a) and transverse (b) views of reconstructed native arterial geometries for P1, and longitudinal (c) and transverse (d) views of reconstructed native arterial geometries for P2. Reconstructions of bones in the arm and hand are included as a reference in (a) and (c).

Native arterial geometries from two “patients,” henceforth referred to as P1 and P2, were reconstructed using Computed Tomography (CT) scan data of the left arm and hand. The anonymized CT datasets were obtained retrospectively from adult subjects who had undergone clinically-indicated upper-body CT angiography in the setting of major trauma and were found to have normal vasculature. Specifically, the P1 dataset was collected using a Philips Brilliance 64 Helical CT scanner (841 slices in total with a voxel size of 0.91 mm × 0.91 mm × 1 mm), and the P2 dataset was collected using a Philips ICT 256 Helical CT scanner (372 slices in total with a voxel size of 0.60 mm × 0.60 mm × 2 mm). The data collection had local research governance approval.

Segmentation of the CT datasets was undertaken with ITK Snap 2.4.0,^63^ using a semi-automatic approach for reconstructing the brachial artery and the proximal sections of the radial and ulnar arteries, and manual segmentation for reconstructing the distal sections of the radial and ulnar arteries. Vessel surfaces were then extracted using VMTK,^3^ and smoothed using a combination of VMTK and PTC-Creo Parametric. Specifically, VMTK was used to perform an initial smoothing, from which vessel centrelines were extracted. Variation of maximum inscribed sphere radius was then calculated and averaged in five equisized sections of centreline. Finally, PTC-Creo Parametric swept a circle along each centreline to form the final geometries, where the circle radius was varied smoothly between the averaged maximum inscribed sphere radii in each section of the centreline using the “loft” command, which interpolates using a NURBS surface. Images of the resulting arterial geometries for P1 and P2 are shown in Fig. 1. Reconstructions of bones in the arm and hand are included for reference in Fig. 1.

#### Forming an Arterio-Venous Fistulae

**Figure 2:**
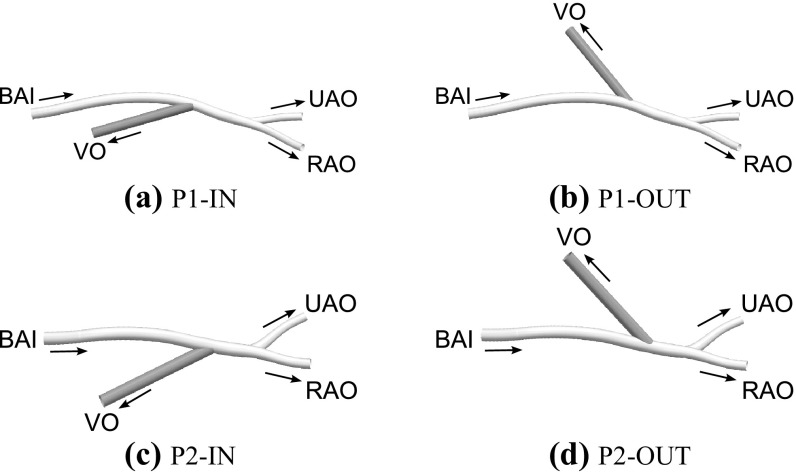
AVF configurations formed by connecting a straight vein, shaded in grey, onto arterial sections of P1 and P2. Specifically, P1-IN (a) is formed by connecting a vein onto the inner curvature of an arterial bend in P1, P1-OUT (b) is formed by connecting a vein onto the outer curvature of an arterial bend in P1, P2-IN (c) is formed by connecting a vein onto the inner curvature of an arterial bend in P2, and P2-OUT (d) is formed by connecting a vein onto the outer curvature of an arterial bend in P2. The Brachial Artery Inlet (BAI), Radial Artery Outlet (RAO), Ulnar Artery Outlet (UAO), and the Venous Outlet (VO) are labelled.

Straight veins were digitally anastomosed onto the inner/outer curvature of an arterial bend in P1/P2 using the CAD software PTC Creo Parametric to create brachiocephalic fistulae. Specifically, following Iori *et al.*,^32^ inner configurations were designed such that the anastomosis was located on the opposite side of the artery to the bulk of any skewed arterial flow (verified to be the case *a posteriori*), and outer configurations were designed such that the anastomosis was located on the same side of the artery as the bulk of any skewed arterial flow (also verified to be the case *a posteriori*). In all cases the vein was anastomosed proximal to the radial/ulnar bifurcation following Chen *et al.* 18 Moreover, in line with various previous studies,^11^,^24^,^31^,^32^,^57^ the vein had a diameter equal to that of the BAI and made an angle of 35° with the local centre-line of the native artery. In all cases the native arterial geometry was cropped approximately $6\times 10^{-2}$ m upstream and downstream of the anastomosis. The resulting brachiocephalic AVF configurations for P1 and P2 are shown in Fig. 2.

### Governing Equations

#### Blood Flow

For all configurations blood was treated as an incompressible Newtonian fluid. Specifically, blood flow was modeled using the time-dependent incompressible 3D Navier–Stokes equations for a fluid with constant viscosity, which can be written as

1 $$\varvec{\nabla }\cdot \mathbf{u}={\rm 0},$$

2 $$\rho \frac{\partial {\mathbf{u}}}{\partial {t}}+ \rho \mathbf{u}\cdot {\varvec{\nabla }}\mathbf{u}+{\varvec{\nabla }}p=\mu {\varvec{\nabla }}^2\mathbf{u},$$

where $\mu$ is the viscosity of human blood, $\rho$ is the density of human blood, $\mathbf{u}$ is the three-dimensional blood velocity (vector) field, and *p* is the pressure. Values of $\mu =3.5\times 10^{-3}$ Pa s and $\rho =1060$ kg m^−3^ 17 were employed.

The assumption of Newtonian rheology ignores the well known “shear-thinning” property of blood, which begins to occur at shear rates below 50–100 s^−1^ 40,44,62 and is particularly significant below shear rates of 10 s^−1^.^40^ An *a posteriori* analysis of results from P1-OUT revealed up to 100, 99 and $93\%$ of the total blood volume was exposed to a shear rate above 10, 100 and 250 s^−1^, respectively, at some stage during a single pulse, with analogous figures of 100, 98 and $83\%,$ respectively, obtained for P2-OUT. These significant exposures to high shear, at some stage during a single pulse, suggest widespread suppression of rouleaux formation, which is responsible for shear-thinning and occurs on a time-scale of order 1 s.^51^ Consequently, the assumption of Newtonian rheology is considered reasonable. We also note that the assumption has been widely used in previous studies of blood flow in AVF and vascular grafts.^9^,^11^,^32^,^34^,^38^,^43^,^54^

#### Oxygen Transport

For all configurations oxygen was treated as a passive scalar dissolved in blood plasma. Specifically, oxygen transport was modeled using the time-dependent 3D advection-diffusion equation, which can be written as

3 $$\frac{\partial C}{\partial t}=\kappa \varvec{\nabla} ^2C-\mathbf{u}\cdot \varvec{\nabla} {\rm C},$$

where $\kappa$ is the diffusivity of oxygen in human plasma, $\mathbf{u}$ is the three-dimensional blood velocity (vector) field, and *C* is the oxygen concentration. A value of $\kappa =1.2\times 10^{-9}$ m$^{2}$ s^−1^ 19 was used in this study.

The assumption that oxygen is a passive scalar dissolved in blood plasma ignores the effect of haemoglobin, to which oxygen can bind. However, previous studies suggest that haemoglobin simply acts to augment oxygen transport patterns by a spatially constant factor of approximately two,^37^,^45^ which can be accounted for *a posteriori* when the results are analysed (see Section “Wall Shear Stress and Wall Normal Oxygen Flux”).

### Boundary Conditions

#### Blood Flow

**Figure 3:**
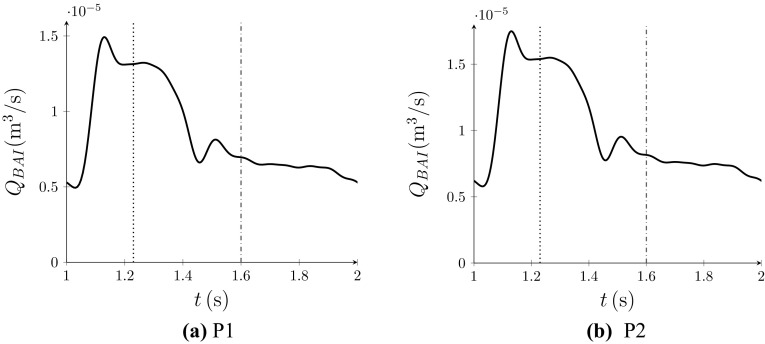
$Q_{\textit{BAI}}$ for P1 (a) and P2 (b). The dotted and dash-dotted vertical lines indicate time points referenced from Fig. 5.

A no-slip boundary condition was applied at the AVF wall, which was considered to be rigid. A space–time varying velocity boundary condition was imposed at the BAI of each AVF configuration. The inflow rate waveform, which had a period of 1 s, was taken from Sigovan *et al.* 54 Specifically the first 15 Fourier modes were extracted, and the signal was scaled such that the peak Reynolds number at the BAI was 1300, in agreement with Sigovan *et al.* 54 The resulting time-averaged Reynolds number at the BAI, $Re_{\textit{BAI}},$ was $\sim$750, similar to the time-constant inflow Reynolds number used in Iori *et al.* 32 All flow was prescribed normal to the BAI inflow plane, and following previous studies,^28^,^34^,^43^ a Womersley profile was imposed in space. The inflow rate waveforms at the BAI $Q_{\textit{BAI}}$ for P1 and P2 are shown in Fig. 3.

An RCR Windkessel model was applied at the RAO, UAO and VO. Specifically

4 $$P_{\textit{RAO}}=Q_{\textit{RAO}}(R_{1{\textit{RAO}}}+R_{2{\textit{RAO}}})-R_{2{\textit{RAO}}}C_{\textit{RAO}}\frac{{d}}{{d}t}(P_{\textit{RAO}}-R_{1{\textit{RAO}}}Q_{\textit{RAO}}),$$

5 $$P_{\textit{UAO}}=Q_{\textit{UAO}}(R_{1\textit{UAO}}+R_{2\textit{UAO}})-R_{2\textit{UAO}}C_{\textit{UAO}}\frac{{d}}{{d}t}(P_{\textit{UAO}}-R_{1\textit{UAO}}Q_{\textit{UAO}}),$$

6 $$P_{\textit{VO}}=Q_{\textit{VO}}(R_{1\textit{VO}}+R_{2\textit{VO}})-R_{2\textit{VO}}C_{\textit{VO}}\frac{{d}}{{d}t}(P_{\textit{VO}}-R_{1\textit{VO}}Q_{\textit{VO}}),$$

where $P_{\textit{RAO}},$ $P_{\textit{UAO}}$ and $P_{\textit{VO}}$ are the spatially averaged pressure waveforms at the RAO, UAO, and VO respectively, $Q_{\textit{RAO}},$ $Q_{\textit{UAO}}$ and $Q_{\textit{VO}}$ are the outflow rate waveforms at the RAO, UAO, and VO respectively, and $R_{1{\textit{RAO}}},$ $R_{2{\textit{RAO}}},$ $C_{\textit{RAO}},$ $R_{1\textit{UAO}},$ $R_{2\textit{UAO}},$ $C_{\textit{UAO}},$ $R_{1\textit{VO}},$ $R_{2\textit{VO}},$ $C_{\textit{VO}}$ are relevant Windkessel parameters.

Values for the Windkessel parameters were selected using an approach similar to Pant *et al.* 47 Specifically, they were obtained for P1-IN and P2-IN configurations using an iterative approach, that aimed to minimise

7 $$\Upphi =\left( \frac{\max (P_{\textit{BAI}})-P_{RS}}{P_{RS}}\right) ^2+\left( \frac{\min (P_{\textit{BAI}})-P_{RD}}{P_{RD}}\right) ^2+\left( \frac{{\int }|Q_{\textit{VO}}-Q_{R}|\;{d}t}{{\int }|Q_{R}|\;{d}t}\right) ^2,$$

where $P_{\textit{BAI}}$ is the pressure waveform at the BAI, $P_{RS}=130$ mmHg and $P_{RD}=80$ mmHg are reference systolic and diastolic pressures, respectively, at the BAI taken from Sigovan *et al.*,^54^ $Q_{R}$ is a reference outflow rate waveform at the VO taken from Sigovan *et al.*,^54^ and the integrals are taken to be over a single pulse, when the solution has become period independent.

**Figure 4:**
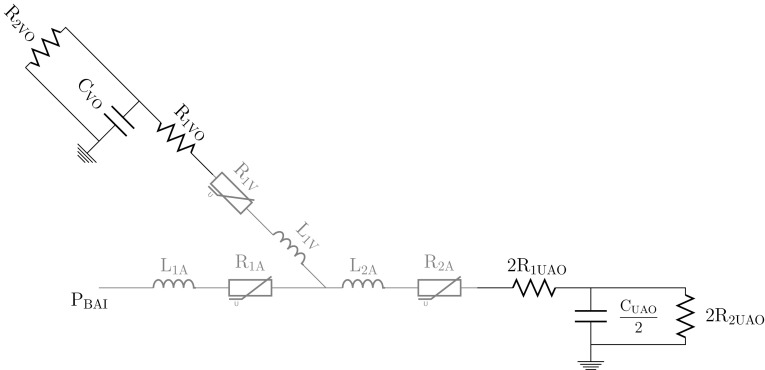
Schematic illustration of the 0D model used in the Windkessel parameter estimation process. Grey components were used to represent the low-resolution 3D model.

The iterative process itself employed a 0D lumped parameter model, detailed in Fig. 4, combined with a low-resolution time-dependent 3D model of flow in each configuration. The 0D model represented the low-resolution 3D model in terms of three inductors, with inductances $L_{1A},$ $L_{2A},$ and $L_{1V}$ as per Fig. 4 as well as three quadratically non-linear resistors with resistances $R_{1A},$ $R_{2A},$ and $R_{1V}$ as per Fig. 4. The 3D model solved the governing flow equations as per Section “Blood Flow” using StarCCM+ v9.06.9 as per Section “Computational Method”, but with ${\sim }1/11$ of the spatial resolution and 1 / 5 of the temporal resolution. It was assumed that the UAO and RAO has the same peripheral behavior i.e., $R_{1\textit{UAO}}=R_{1{\textit{RAO}}},$ $R_{2\textit{UAO}}=R_{2{\textit{RAO}}}$ and $C_{\textit{UAO}}=C_{\textit{RAO}}.$ Hence, both outlets were merged in the 0*D* model as per Fig. 4, and were parameterized in terms of $R_{1\textit{UAO}},$ $R_{2\textit{UAO}}$ and $C_{\textit{UAO}}$ alone.

To begin the iterative process, $L_{1A},$ $L_{2A},$ $L_{1V},$ $R_{1A},$ $R_{2A},$ and $R_{1V}$ were estimated, and the 0D model was used to identify values of the Windkessel parameters that minimized $\Upphi .$ These parameters were then used to define boundary conditions for the low-resolution 3*D* model, which was run until the solution became period independent, and from which improved estimates for $L_{1A},$ $L_{2A},$ $L_{1V},$ $R_{1A},$ $R_{2A},$ and $R_{1V}$ were then extracted. These were fed back into the 0*D* model, and the process was repeated until the Windkessel parameters were seen to converge.

Values of the Windkessel parameters obtained for the P1-IN and P2-IN simulations are shown in Table 1. These parameters were also used for P1-OUT and P2-OUT simulations, respectively.

**Table 1:** Windkessel parameters obtained for the P1-IN and P2-IN simulations.

	P1-IN	P2-IN
$R_{1{\textit{RAO}}}$ (mmHg mL^−1^s)	6.88	3.20
$R_{2{\textit{RAO}}}$ (mmHg mL^−1^s)	59.80	53.20
$C_{\textit{RAO}}$ (mL (mmHg)^−1^)	0.010	0.009
$R_{1\textit{UAO}}$ (mmHg mL^−1^s)	6.88	3.20
$R_{2\textit{UAO}}$ (mmHg mL^−1^s)	59.80	53.20
$C_{\textit{UAO}}$ (mL (mmHg)^−1^)	0.010	0.009
$R_{1\textit{VO}}$ (mmHg mL^−1^s)	7.00	7.00
$R_{2\textit{VO}}$ (mmHg mL^−1^s)	10.30	7.50
$C_{\textit{VO}}$ (mL (mmHg)^−1^)	10.50	5.25

The parameters used for P1-IN and P2-IN simulations were also used for P1-OUT and P2-OUT simulations, respectively

The resulting average flow splits between the VO and the distal arteries in a temporal window spanning the full pulse period (henceforth referred to as WT), systole from 1.1 to 1.4 s (henceforth referred to as WS), and part of diastole from 1.4 to 1.7 s (henceforth referred to as WD), were ${\sim }66{:}34,$ ${\sim }60{:}40,$ and ${\sim }78{:}22$ respectively.

The resulting pressure at the BAI varied in the range 82–123 mmHg for P1-IN, and 80–132 mmHg for P2-IN, with similar ranges for P1-OUT and P2-OUT, respectively. The average pressure-drop between the BAI and the VO was $\sim$4 mmHg for both P1-IN and P2-IN, with a similar value for both P1-OUT and P2-OUT. The maximum pressure-drop between the BAI and the VO was $\sim$11 mmHg for P1-IN, and $\sim$9 mmHg for P2-IN, with similar values for P1-OUT and P2-OUT, respectively.

We note that a Dean number *De* defined as $De=Re_{\textit{BAI}}\sqrt{\kappa _A D_A/2},$ where $\kappa _A$ and $D_A$ are the local arterial curvature and arterial diameter respectively at a given point in the artery, never exceeds 320 anywhere in the proximal arterial section of any configuration i.e., it is always well below the critical Dean number of ${\sim}$900 at which multiple unsteady vortices develop in a planar curved tube.^21^

#### Oxygen Transport

Following Iori *et al.*,^32^ a steady-state spatially-constant oxygen concentration of $C_{\textit{NBAI}}=1.305\times 10^{-1}$ mol m^−3^ was applied at the BAI (based on an oxygen partial pressure of 75 mmHg^12^ and a Henry’s law constant of $1.74\times 10^{-3}$ mol m^−3^ mmHg^−1^ 35). Zero boundary-normal oxygen concentration gradients were applied at the VO, RAO, and UAO. Also, for all configurations a steady-state spatially-constant oxygen concentration of $C_{W}=1.044\times 10^{-1}$ mol m^−3^ was applied at the arterial and venous walls (based on an oxygen partial pressure of 60 mmHg^12^ and a Henry’s law constant of $1.74\times 10^{-3}$ mol m^−3^ mmHg^−1^ 35).

The imposition of a spatially-constant oxygen concentration at the proximal arterial inlet is based on the assumption that oxygen is “well mixed” upstream of the domain. The imposition of a spatially-constant oxygen concentration at the arterial and venous walls is based on the assumption that the walls act as an oxygen sink, readily consuming oxygen.^58^

### Computational Method

Polyhedral unstructured volume meshes, with prismatic boundary layer meshes adjacent to the arterial wall, were constructed for each configuration. The meshes were refined near the anastomosis. Specifically, elements near the anastomosis had an average size of ${\sim }6\times 10^{-5}$ m, expanding progressively to ${\sim }1.5\times 10^{-4}$ m beyond a distance of ${\sim }0.045$ m from the anastomosis. The prismatic boundary layer meshes were 25 elements thick, with the first element having a thickness of less than $3\times 10^{-6}$ m; in line with the mesh resolution employed in Coppola and Caro.^19^ Each mesh had ${\sim }11\times 10^{6}$ elements in total. Results of a mesh independence study are presented in Appendix A.

Solutions for the blood velocity field and the oxygen concentration were obtained using Star-CCM+ v9.06.9 *via* the following procedure:

- Each simulation was initialized with zero velocity, zero oxygen concentration, and a pressure of 78 mmHg, and run with the coupled steady solver until every momentum and mass continuity residual fell below $10^{-10}.$
- Each steady-state solution was then used as the initial condition for the coupled implicit unsteady solver, which advanced solutions 1 s in time (1 pulse period). For all simulations a timestep of $1\times 10^{-4}$ s was used.
- Each simulation was then advanced a further 1 s (1 pulse period) using the coupled implicit unsteady solver, during which time data were exported for analysis. Results of a period independence study are presented in Appendix B. For all simulations a timestep of $1\times 10^{-4}$ s was used.

Each simulation was carried out on 64 cores of a Dell AMD Opteron 64-core server with 512 GB RAM, and required approximately 4 weeks to complete.

## Results

### Unsteady Analysis

#### Qualitative Insight

**Figure 5:**
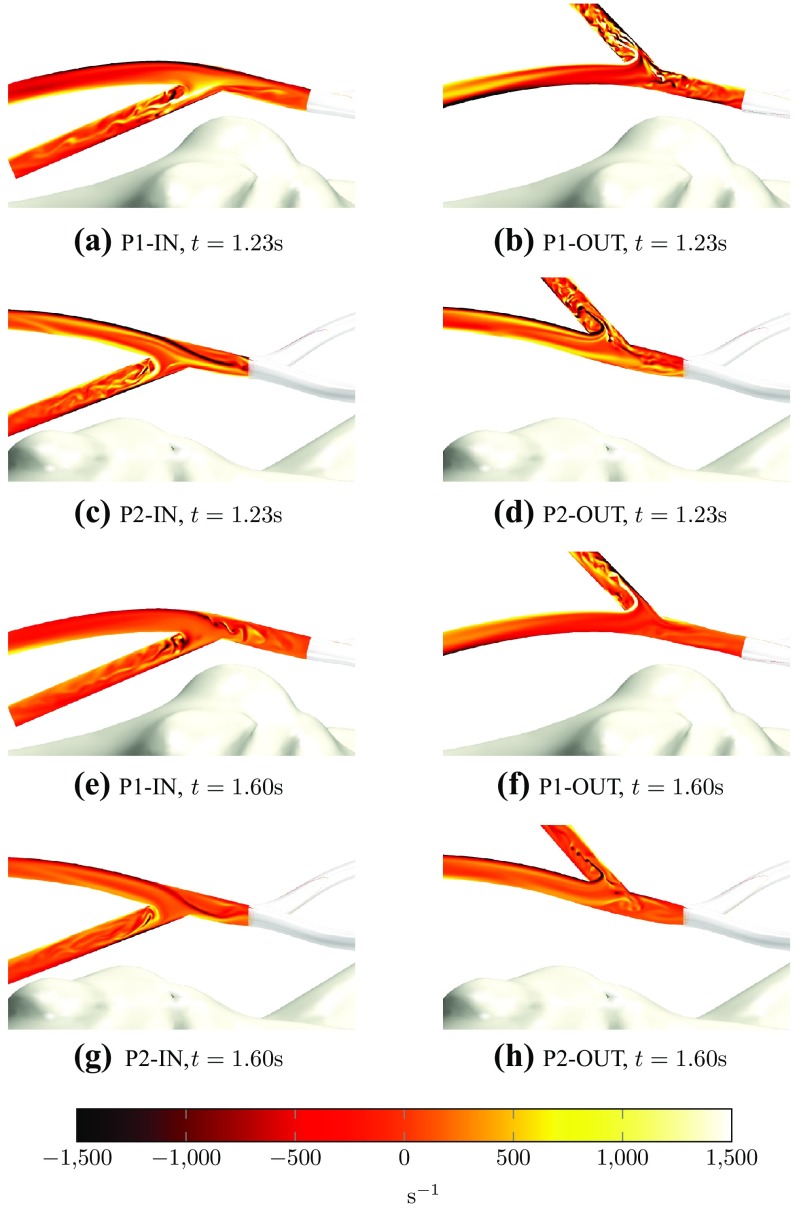
Snapshots of plane-normal vorticity on planes within each AVF configuration at 1.23 s, which is during systole (marked with vertical dotted lines in Fig. 3), and at 1.60 s, which is during diastole (marked with vertical dashed-dotted lines in Fig. 3).

Figure 5 shows snapshots of plane-normal vorticity on planes within each AVF configuration during systole and diastole.

Unsteady flow can be observed in the venous section of all configurations at almost all points in the pulse cycle. This result is in line with the idealized model of Iori *et al.*,^32^ which predicted unsteady venous flow when the diameter of the artery and vein were the same. However, converse to the idealized model of Iori *et al.*,^32^ unsteady vortex shedding is also present in arterial sections when the vein is connected to the outer curvature of the arterial bend (P1-OUT and P2-OUT). In particular, within WS during systole, flow in the arterial sections of P1-OUT and P2-OUT appear to be more unstable than flow in the arterial sections of P1-IN and P2-IN. However, within WD during diastole, the picture is less clear. Converse to the results from WS, it appears that P1-IN is relatively more unsteady than P1-OUT. However, results from P2-IN and P2-OUT are similar.

#### Quantitative Insight

**Figure 6:**
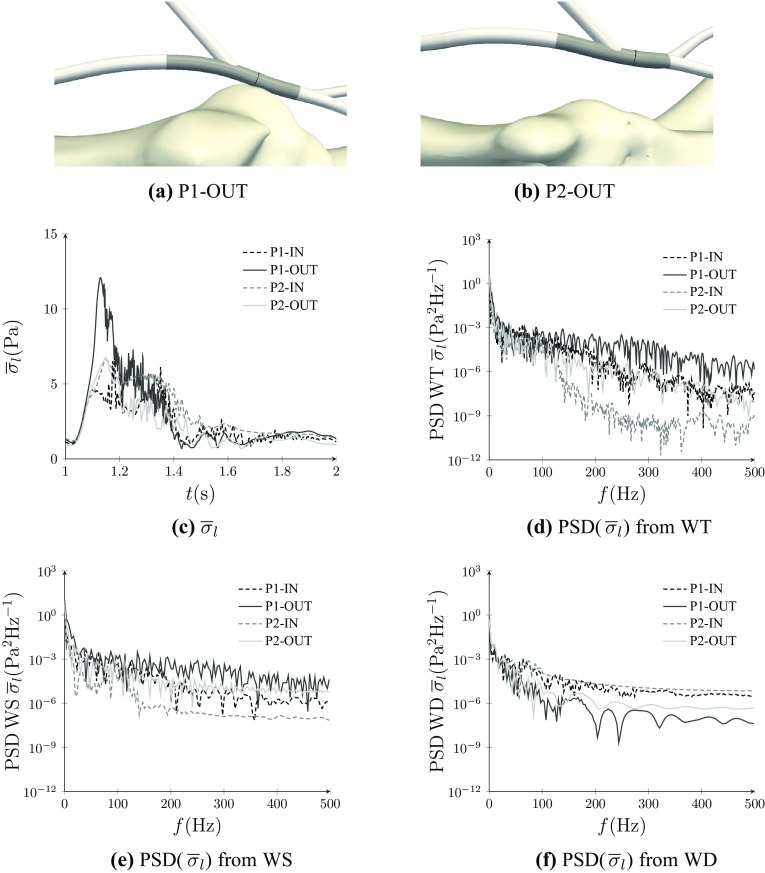
Views of P1-OUT (a) and P2-OUT (b) with rings of arterial sections from which $\overline{\sigma }_{\rm l}$ were extracted for spectral analysis marked in black, and regions of arterial sections from which ${\varvec{\sigma }}-\overline{{\varvec{\sigma }}}_t$ were extracted for POD analysis shaded in grey. Plots of $\overline{\sigma }_{\rm l}$ for each configuration (c), PSD from WT (spanning the full pulse period) of $\overline{\sigma }_{\rm l}$ for each configuration (d), PSD from WS (spanning systole) of $\overline{\sigma }_{\rm l}$ for each configuration (e), and PSD from WD (spanning part of diastole) of $\overline{\sigma }_{\rm l}$ for each configuration (f).

Temporal variation of $\overline{\sigma }_{\rm l},$ the WSS magnitude averaged over a ring of arterial section (see Fig. 6), was extracted from each simulation. Plots of $\overline{\sigma }_{\rm l}$ for each AVF configuration are shown in Fig. 6 together with the Power Spectral Density (PSD) extracted from WT, WS, and WD.

It is clear that the WSS in arterial sections of all AVF configurations exhibit high-frequency fluctuations. This unsteadiness appears to be irrespective of whether the venous section of the AVF is connected to the inner or outer curvature of an arterial bend; converse to the idealized model of Iori *et al.* 32 which predicted that connecting the venous section of the AVF to the outer curvature of an arterial bend would suppress unsteadiness in the artery. Further observations can also be made. Firstly, within WS during systole, P1-OUT and P2-OUT appear to have relatively more energy in higher frequency modes than P1-IN and P2-IN respectively. This results is in line with the qualitative observations of Section “Qualitative Insight.” However, within window WD during diastole, the picture is less clear. Converse to the results from WS, it appears that P1-IN and P2-IN have relatively more energy in higher frequency modes than P1-OUT and P2-OUT respectively, but the differences are less distinct.

Further quantitative assessment of unsteadiness was undertaken using snapshot Proper Orthogonal Decomposition (POD)^28^,^41^,^55^ of ${\varvec{\sigma }}-\overline{{\varvec{\sigma }}}_t,$ where ${\varvec{\sigma }}$ is the space–time WSS vector field and $\overline{{\varvec{\sigma }}}_t$ is the temporal average of the space–time WSS vector field, in an arterial section of each AVF configuration near the anastomosis (see Fig. 6). Specifically, snapshot POD was undertaken in WT, WS, and WD. For WT, 1000 temporal snapshots with a uniform spacing of 0.001 s were used. For WS and WD, 1500 temporal snapshots with a uniform spacing of 0.0002 s were used.

The fraction of POD modes required to reconstruct $96\%$ of the energy content of each ${\varvec{\sigma }}-\overline{{\varvec{\sigma }}}_t$ is given in Table 2. Taking this fraction as a proxy for the range of energized frequencies and overall unsteadiness, various observations can be made. Firstly, within WS, during systole, P1-OUT and P2-OUT appear to be relatively more unsteady than P1-IN and P2-IN respectively. This results is in line with the qualitative observations of Section “Qualitative Insight” and the quantitative analysis of Section “Quantitative Insight.” However, within window WD, during diastole, the picture is less clear. Converse to the results from WS, it appears that P1-IN is relatively more unsteady than P1-OUT. However, P2-IN is relatively only slightly more unsteady than P2-OUT.

**Table 2:** Number of modes needed to reconstruct 96$\%$ of the energy content of each ${\varvec{\sigma }}-\overline{{\varvec{\sigma }}}_t$ in WT, WS, and WD.

	WT	WS	WD
P1-IN	19/1000	26/1500	45/1500
P1-OUT	7/1000	50/1500	11/1500
P2-IN	5/1000	17/1500	11/1500
P2-OUT	6/1000	19/1500	10/1500

#### Summary

The main finding of the above unsteady analysis is that, converse to results from previous more idealized studies,^32^ forming an AVF by connecting a vein onto the outer curvature of an arterial bend does not, necessarily, suppress unsteady flow in the artery. Specifically, during systole, outer configurations are actually more unstable than inner configurations, and whilst this appears somewhat reversed in diastole, the situation is less clear, and all configurations exhibit a degree of unsteadiness.

We note that during WS, in systole, the average flow split between the vein and the distal arteries is ${\sim }60{:}40.$ This is significantly more even than the fixed flow split of 80 : 20 prescribed by Iori *et al.* 32 We can speculate that it is this, more even flow split during WS, that causes the outer configurations to become unstable; noting in particular from Fig. 5 that during WS the outer configurations become unstable in arterial sections distal to the anastomosis, when/where the current simulations exhibit a relatively higher flow rate *cf.* Iori *et al.* 32 This assertion is further supported by the fact that during WD, in diastole, the average flow split between the vein and the distal arteries is ${\sim }78{:}22,$ and the outer configurations recover a degree of stability—although not enough to overcome the unsteadiness induced during WS.

### Time-Averaged Analysis

#### Wall Shear Stress and Wall Normal Oxygen Flux

**Figure 7:**
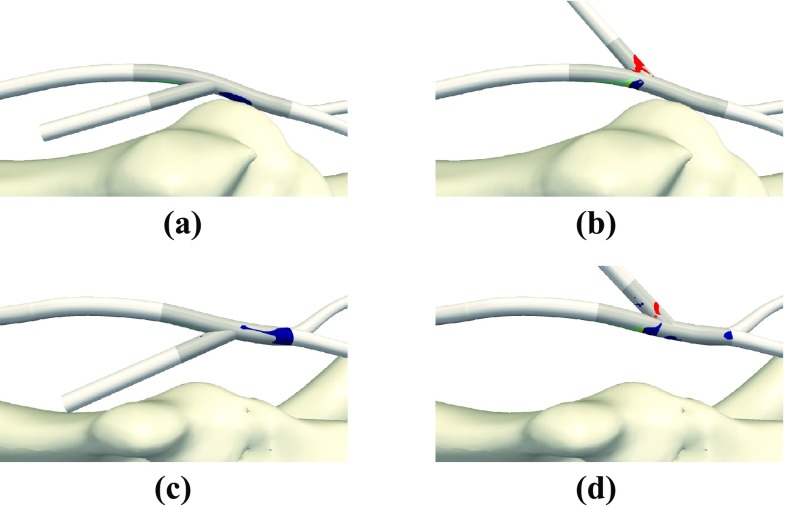
Quad-color maps of WSS^−^ (blue), WSS^+^ (red), LWNOF^−^ (green), and the intersection between regions of WSS^−^ and LWNOF^−^ (yellow) in arterial and venous sections of P1-IN (a), P1-OUT (b), P2-IN (c), P2-OUT (d).

**Figure 8:**
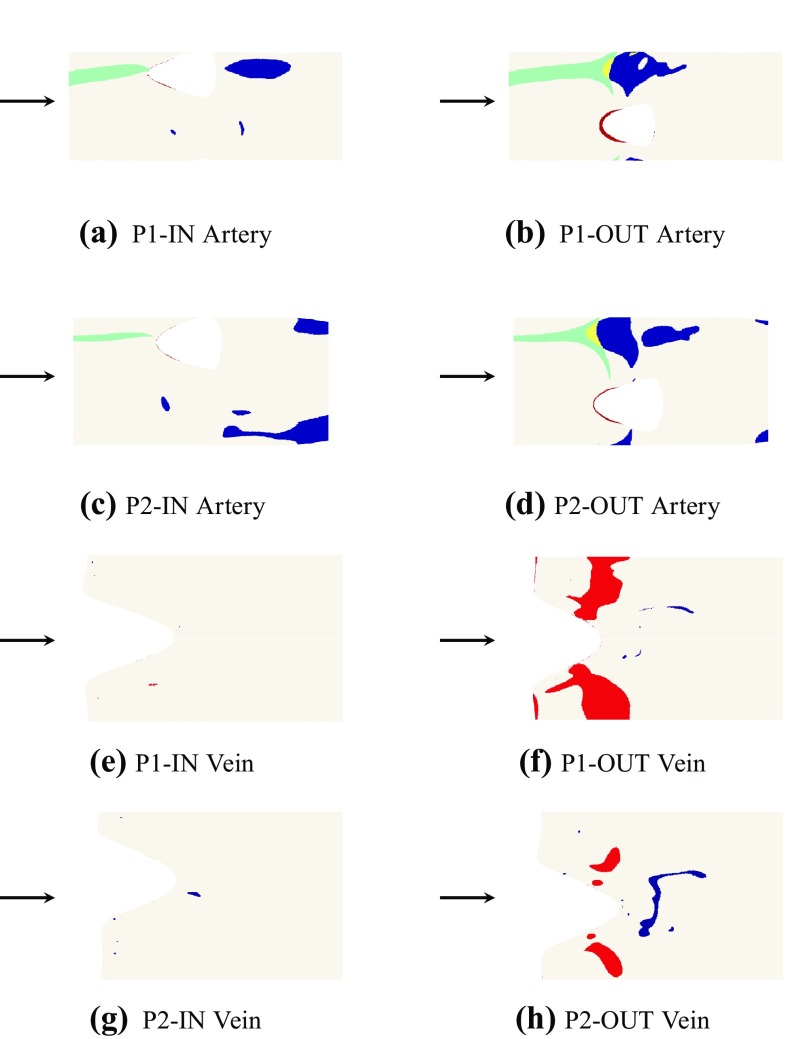
Quad-color maps of WSS^−^ (blue), WSS^+^ (red), LWNOF^−^ (green), and the intersection between regions of WSS^−^ and LWNOF^−^ (yellow) in excised and flattened arterial sections of Fig. 7 of P1-IN (a), P1-OUT (b), P2-IN (c), P2-OUT (d) and in excised and flattened venous sections of Fig. 7 of P1-IN (e), P1-OUT (f), P2-IN (g), P2-OUT (h). Arrow indicates direction of blood flow. The white holes mark the location of the venous anastomoses.

**Figure 9:**
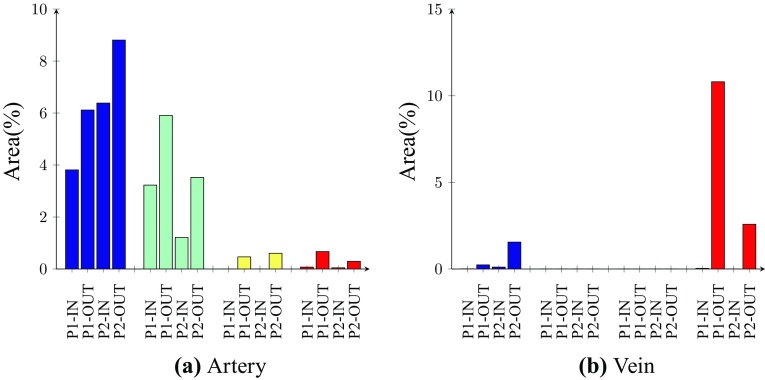
Bar chart showing the percentage area of WSS^−^ (blue), WSS^+^ (red), LWNOF^−^ (green), and the intersection between regions of WSS^−^ and LWNOF^−^ (yellow), in excised and flattened arterial sections of each configuration (a) and in excised and flattened venous sections of each configuration (b).

Quad-color maps of WSS^−^ (“pathologically low” time-averaged WSS magnitude <0.5 Pa), WSS^+^ (“pathologically high” WSS magnitude >30 Pa), LWNOF^−^ (“pathologically low” time-averaged LWNOF $<4.275\times 10^{-7}$ mol m^−2^ s^−1^), and the intersection between regions of WSS^−^ and LWNOF^−^ are shown in Fig. 7, and the corresponding excised and flattened arterial and venous sections from each AVF configuration are shown in Fig. 8. Bar charts showing the percentage area of WSS^−^, WSS^+^, LWNOF^−^, and the intersection between regions of WSS^−^ and LWNOF^−^ in the excised and flattened arterial and venous sections of each configuration are shown in Fig. 9. Note that LWNOF is defined here as $-2\kappa {\mathbf{n}}\cdot {\varvec{\nabla }}c,$ where ${\mathbf{n}}$ is the outward facing wall normal and the factor of two accounts for the role of hemoglobin.^37^,^45^

Following Iori *et al.*,^32^ the pathologically low WSS threshold of 0.5 Pa was chosen to be at the lower-bound of estimates made by various authors, including Masuda *et al.* 42 and Sho *et al.* 52,53 who suggest 0.5 Pa (in rabbits), Dolan *et al.* 23 who suggest 1 Pa, and Irace *et al.* 33 who suggest 1.8 Pa. The pathologically high WSS threshold of 30 Pa was chosen to be at the upper-bound of estimates made by various authors, including Dolan *et al.* 23 who suggest 10 Pa, and Dolan *et al.* 22 who suggest up to 30 Pa. The pathologically low LWNOF threshold was obtained by assuming that the innermost region of the vascular wall (i.e., the intima and media) receive oxygen solely from luminal blood^12^,^29^ and not from the adventitial vasa vasorum. With the further assumptions that this innermost region requires oxygen at a rate of at least $8.55\times 10^{-3}$ mol m^−3^ s^−1^ in order to avoid hypoxia (based on measurements of oxygen consumption in smooth muscle cells of dog femoral arteries^48^), and that it has a thickness of $5\times 10^{-5}$ m (based on measurements in dog femoral arteries^15^), one obtains the threshold of $4.275\times 10^{-7}$ mol m^−2^ s^−1^.

It can be seen that for the P1-OUT and P2-OUT configurations the artery is exposed to significantly more WSS^−^ than for the P1-IN and P2-IN configurations, respectively. Also, for the P1-OUT and P2-OUT configurations, a triangular shaped region of LWNOF^−^ is present in the artery opposite the anastomosis, where stenosis in AVF have previously been observed,^56^ as is a region of overlap between WSS^−^ and LWNOF^−^. Neither of these features are present for the P1-IN and P2-IN configurations. These findings are in agreement with results of previous more idealized studies,^32^ and add further support to the assertion that connecting a vein to the outer curvature of an arterial bend may suppress IH *if* it is caused by low WSS and/or low LNWOF (leading to wall hypoxia). Finally, for the P1-OUT and P2-OUT configuration the vein is exposed to significantly more WSS^+^ than for the P1-IN and P2-IN configurations. Previous more idealized studies, where the vein and artery had the same diameter,^32^ exhibited the same trend. However, the difference in this study between inner and outer curvature configurations was far more marked.

#### Summary

The main finding of the above time-averaged analysis is that, in agreement with results from previous more idealized studies, forming an AVF by connecting a vein onto the inner curvature of an arterial bend will suppress exposure to regions of low WSS and hypoxia in the artery. Results also show that forming an AVF by connecting a vein onto the inner curvature of an arterial bend will significantly reduce exposure to high WSS in the vein.

## Conclusions and Future Work

We have extended previous work by Iori *et al.*,^32^ which investigated the effect of planar arterial curvature on flow and oxygen transport patterns in idealized AVF with non-pulsatile inflow conditions, to more realistic non-planar brachiocephalic AVF configurations with pulsatile inflow conditions. Results from our more realistic simulations show that forming an AVF by connecting a vein onto the outer curvature of an arterial bend does not, necessarily, suppress unsteady flow in the artery. Specifically, during systole, outer configurations are actually more unstable than inner configurations, and whilst this is somewhat reversed in diastole, the situation is less clear, and all configurations exhibit a degree of unsteadiness. This finding is converse to results from the previous more idealized study of Iori *et al.* 32 However, results also show that forming an AVF by connecting a vein onto the inner curvature of an arterial bend will suppress exposure to regions of low WSS and hypoxia in the artery. This finding is in agreement with results from the previous more idealized study of Iori *et al.* 32 Finally, results show that forming an AVF by connecting a vein onto the inner curvature of an arterial bend will significantly reduce exposure to high WSS in the vein. The results are important, as they suggest that in realistic scenarios arterial curvature can be leveraged to reduce exposure to excessively low/high levels of WSS and regions of hypoxia in AVF. This may in turn reduce rates of IH and hence AVF failure.

Future work should address limitations of the current modeling approach. In particular, the effect of wall compliance should be considered. Previous studies have shown time-averaged WSS patterns to be qualitatively similar between models with rigid/compliant walls.^43^ However, the effect of compliance on unsteady dynamics has yet to be elucidated. Future work should also investigate the effect of geometric variations across an extended cohort of patient specific arterial datasets, as well as the effect of anastomotic connection angle, vein-to-artery diameter ratio, venous curvature, and variations in morphology induced by arm movement and bending. Previous studies suggest that neck movement can significantly alter vascular morphology in a patient specific fashion.^4^,^5^ It is therefore possible that the morphology of an AVF changes when a patient moves or bends their arm. Finally, future work should development both bench-top and *in-vivo* pre-clinical experiments, in order to validate the computational results.

## Figures and Tables

**Figure A.1:**
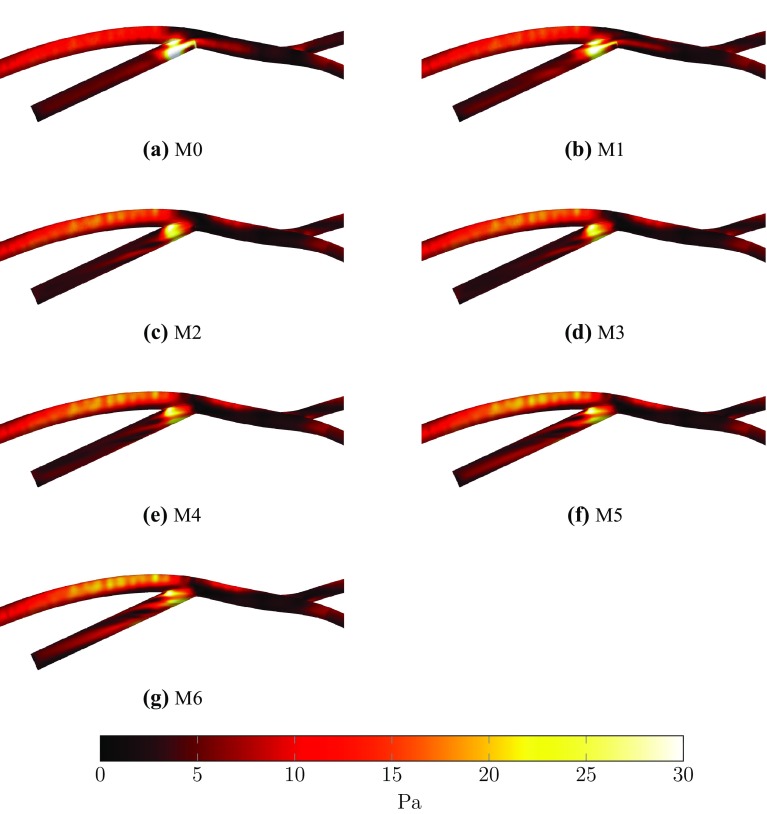
Distributions of $\overline{\sigma }_{\rm t}$ in P1-IN for meshes M0, M1, M2, M3, M4, M5, and M6.

**Figure A.2:**
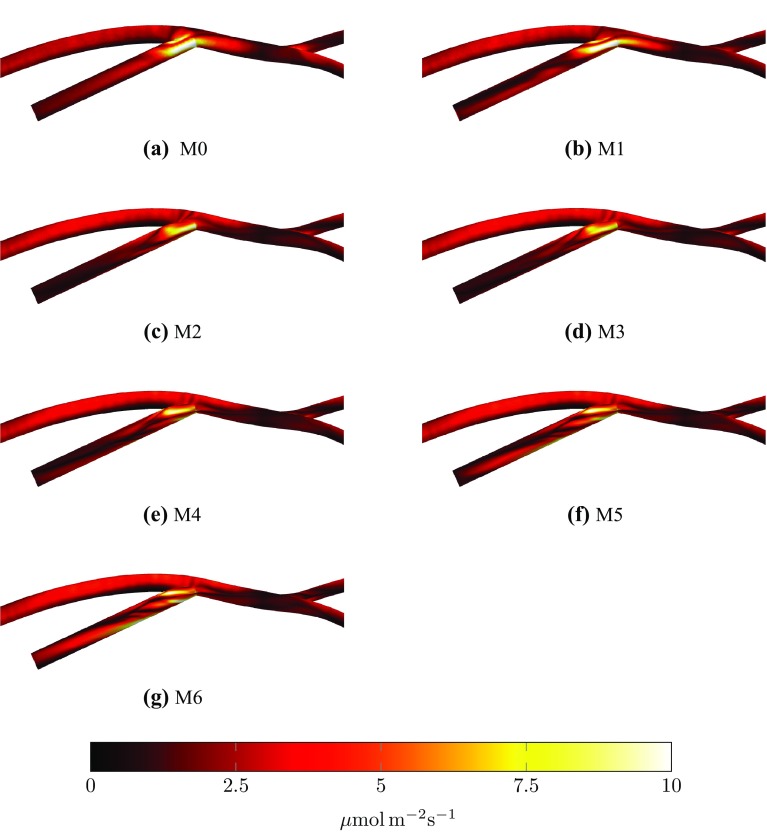
Distributions of $\overline{f}_{\rm t}$ in P1-IN for meshes M0, M1, M2, M3, M4, M5, and M6.

**Figure A.3:**
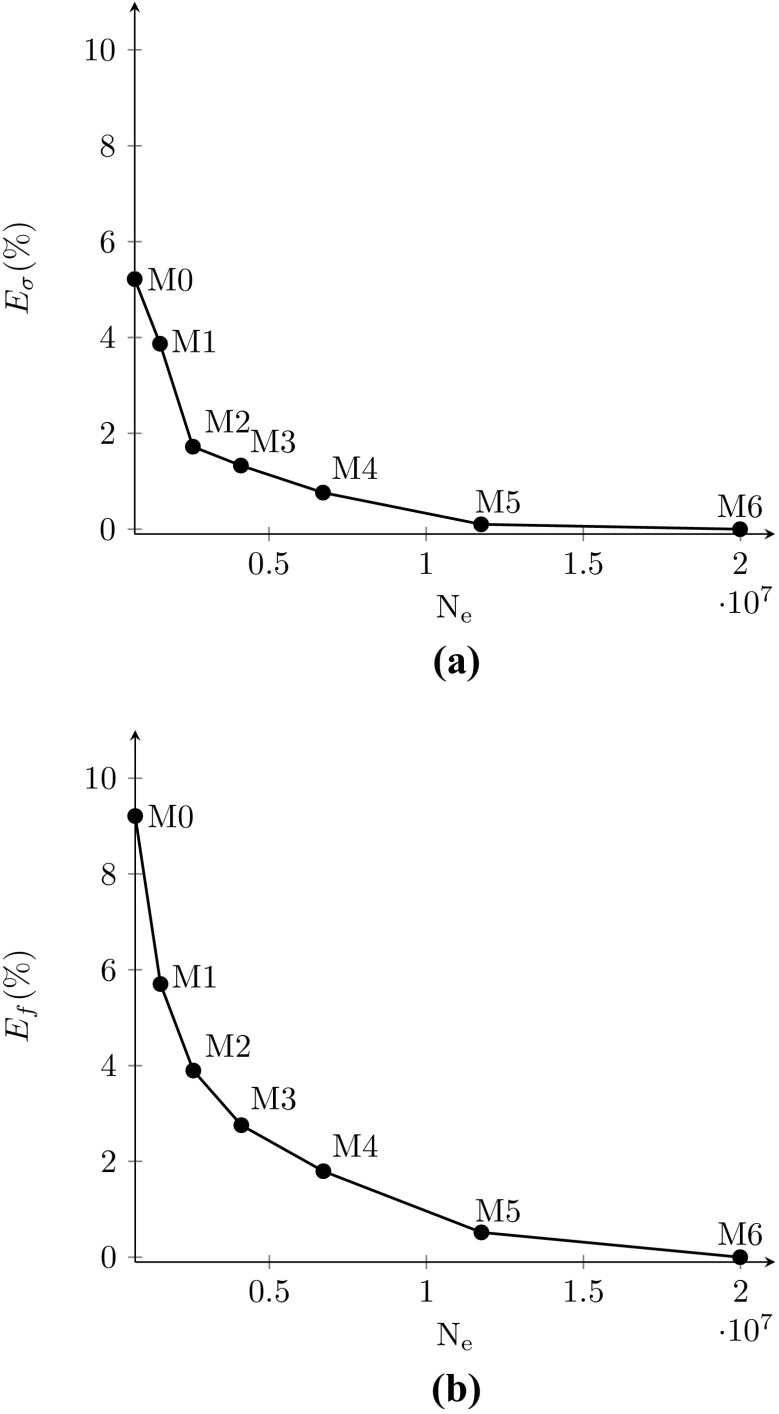
Plots of $E_{\sigma }$ (a) and $E_{\rm f}$ (b) against $N_{\rm e}.$

**Figure A.4:**
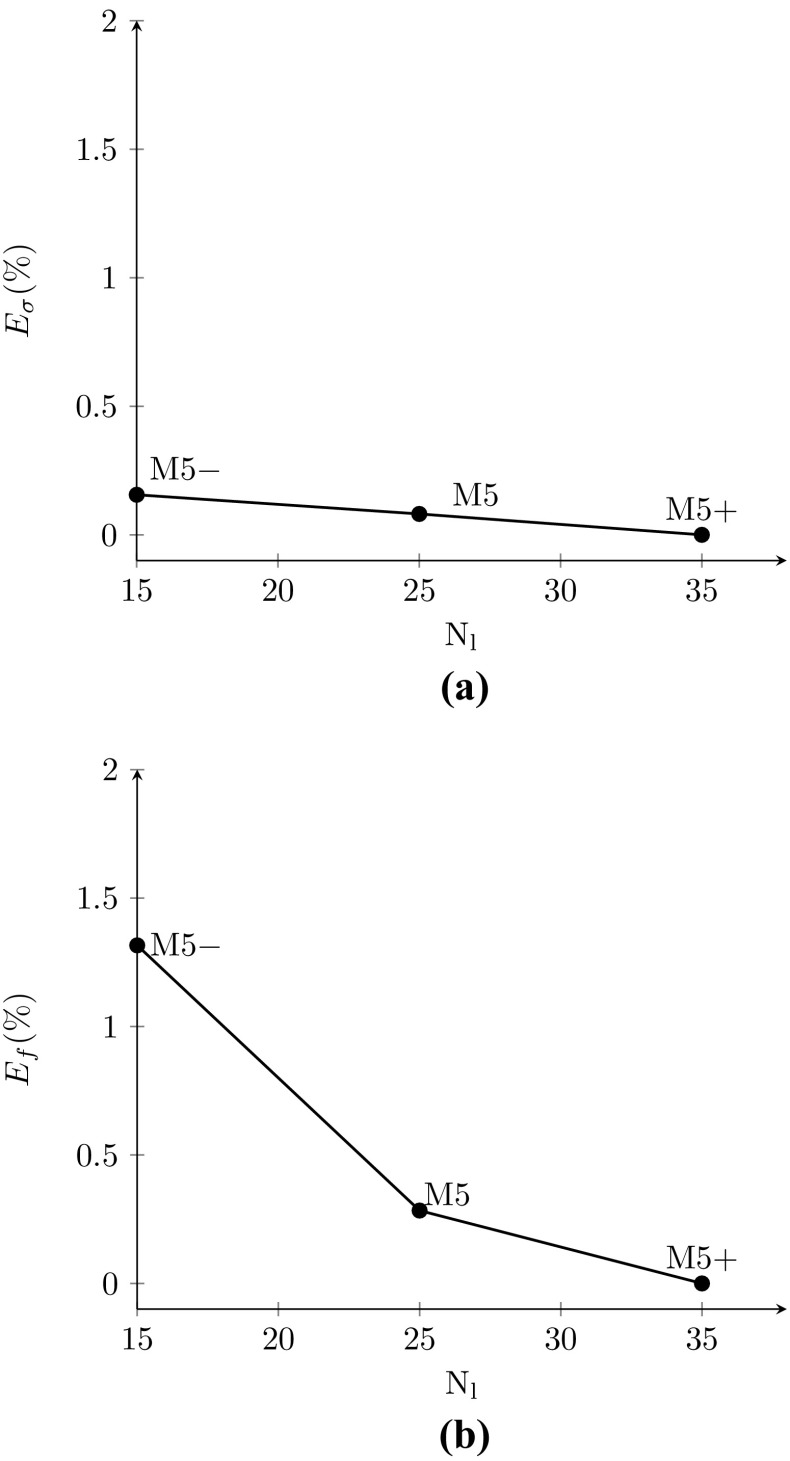
Plots of $E_{\sigma }$ (a) and $E_{\rm f}$ (b) against $N_{\rm l}.$

**Figure B.1:**
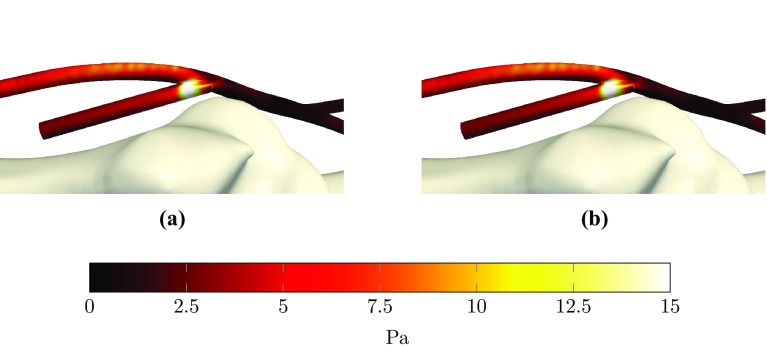
Distributions of $\overline{\sigma }_{\rm t}$ in P1-IN over T1 (a) and T2 (b).

**Figure B.2:**
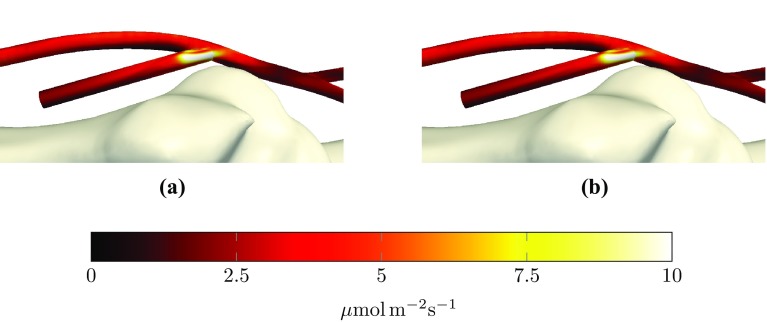
Distributions of $\overline{f}_{\rm t}$ in P1-IN over T1 (a) and T2 (b).

**Figure B.3:**
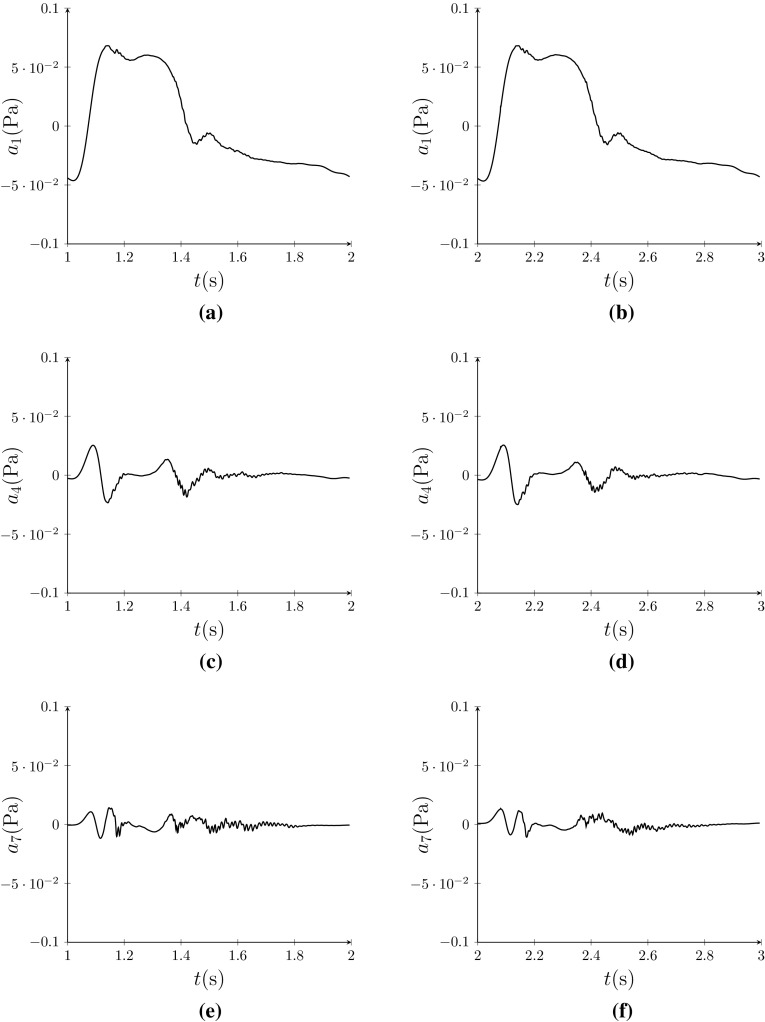
First POD mode *a*_1_ from T1 (a) and T2 (b). Fourth POD mode *a*_4_ from T1 (c) and T2 (d). Seventh POD mode *a*_7_ from T1 (e) and T2 (f).

## Appendix A: Mesh Independence

### Polyhedral Unstructured Volume Mesh

Grid convergence of the polyhedral unstructured volume mesh was assessed by running simulations on seven successively finer discretizations of the P1-IN configuration using Star-CCM+ v9.06.9. All discretizations had a fixed prismatic boundary layer mesh that was 25 elements thick, with the first element having a thickness of ${\sim }2.6\times 10^{-6}$ m; in line with the boundary layer mesh resolution employed in Iori *et al.* 32 For each mesh, the element size near the anastomosis Δ , and the overall number of mesh elements $N_{\rm e},$ are given in Table A.3.

Each simulation had steady inflow boundary conditions. Specifically, the inflow Reynolds number at the BAI was fixed at 1, 300, the flow split between the VO and the distal arteries was 45 : 55, and the flow split between the UAO and the RAO was 50 : 50. These conditions correspond to peak inflow in the associated pulsatile simulation. The oxygen boundary conditions were the same as those used for the pulsatile simulation. Each simulation was initialized by converging to a steady-state solution such that all the residuals had an absolute value smaller than $1\times 10^{-9}.$ Each steady-state solution was then used as the initial condition for the coupled implicit unsteady solver, which advanced solutions 0.2 s, and then a further 0.07 s over which data was extracted for analysis. All unsteady simulation used a time step of $2.2\times 10^{-4}$ s.

Time-averaged WSS magnitude $\overline{\sigma }_{\rm t}$ and time-averaged LWNOF $\overline{f}_{\rm t}$ were extracted from each simulation. Distributions of $\overline{\sigma }_{\rm t}$ and $\overline{f}_{\rm t}$ are shown in Figs. A.1 and A.2 respectively. Space–time-averaged WSS magnitude $\overline{\sigma }_{\rm ts}$ and space–time-averaged LWNOF $\overline{f}_{\rm ts}$ were also calculated for each simulation, and used to obtain percentage errors $E_{\sigma }$ and $E_{\rm f}$ defined as

8 $$E_{\sigma }=\frac{|\overline{\sigma }_{6{\rm ts}}-\overline{\sigma }_{\rm ts}|}{\overline{\sigma }_{6{\rm ts}}}\times 100,$$

and

9 $$E_{\rm f}=\frac{|\overline{f}_{6{\rm ts}}-\overline{f}_{\rm ts}|}{\overline{f}_{6{\rm ts}}}\times 100,$$

where $\overline{\sigma }_{6{\rm ts}}$ and $\overline{f}_{6{\rm ts}}$ are values of $\overline{\sigma }_{\rm ts}$ and $\overline{\sigma }_{\rm ts}$ respectively from mesh M6 (the finest discretization). Plots showing how ${E}_{\sigma }$ and ${E}_{\rm f}$ vary with $N_{\rm e}$ are shown in Fig. A.3.

**Table A.3:** Δ and $N_{\rm e}$ for each mesh.

	Δ (m)	$N_{\rm e}$
Mesh 0	$3.00\times 10^{-4}$	728, 037
Mesh 1	$1.50\times 10^{-4}$	1, 534, 415
Mesh 2	$1.20\times 10^{-4}$	2, 579, 029
Mesh 3	$9.60\times 10^{-5}$	4, 107, 567
Mesh 4	$7.70\times 10^{-5}$	6, 719, 031
Mesh 5	$6.16\times 10^{-5}$	11, 751, 352
Mesh 6	$5.00\times 10^{-5}$	19, 988, 816

### Prismatic Boundary Layer Mesh

Grid convergence of the prismatic boundary layer mesh was assessed by running simulations on versions of Mesh 5 with either increased or decreased boundary layer resolution. Specifically two meshes, henceforth referred to as Mesh $5+$ and Mesh $5-,$ were made. Each mesh had the same overall boundary layer mesh thickness as Mesh 5, but a different number of boundary layer mesh elements $N_{\rm l}$ in the wall normal direction (see Table A.4). Plots showing how ${E}_{\sigma }$ and ${E}_{\rm f}$ vary with $N_{\rm l}$ are shown in Fig A.4.

**Table A.4:** $N_{\rm l}$ and $N_{\rm e}$ for each mesh.

	$N_{\rm l}$	$N_{\rm e}$
Mesh 5−	15	9,456,231
Mesh 5	25	11,751,352
Mesh 5+	35	13,482,563

### Summary

Based on the results presented in Sections ”Polyhedral Unstructured Volume Mesh” and “Prismatic Boundary Layer Mesh,” mesh M5 was used for the simulation in P1-IN, and meshes with similar resolutions were used for simulations in P1-OUT, P2-IN, and P2-OUT.

## Appendix B: Period Independence

The P1-IN simulation was started from a steady-state solution as per Section “Computational Method” and advanced 3 s in time (3 pulse periods) using the coupled implicit unsteady solver and a timestep of $1\times 10^{-4}$ s. Period convergence was assessed by comparing solution data from $t\in [1,2]$ s, hence forth referred to as T1, with solution data from $t\in [2,3]$ s, hence forth referred to as T2.

Distributions of $\overline{\sigma }_{\rm t}$ over T1 and T2 are shown in Fig. B.1. Visually the distributions appear very similar. Quantitatively, spatial averages of $\overline{\sigma }_{\rm t}$ over T1 and T2 were found to be within $0.08\%.$ Distributions of $\overline{f}_{\rm t}$ over T1 and T2 are shown in Fig. B.2. Once again, visually the distributions appear very similar. Quantitatively, spatial averages of $\overline{f}_{\rm t}$ over T1 and T2 were found to be within $0.03\%.$

Unsteady content was compared between T1 and T2 using snapshot POD of ${\varvec{\sigma }}-\overline{{\varvec{\sigma }}}_t$ in an arterial section of each AVF configuration near the anastomosis (see Fig. 6). Specifically, 1000 temporal snapshots with a uniform spacing of 0.001 s were used from each of T1 and T2. Plots of the first, fourth, and seventh temporal POD modes, denoted *a*_1_, *a*_4_, and *a*_7_ respectively, extracted from T1 and T2, are shown in Fig. B.3. Visually the modes appear very similar.

Based on the results presented above, data from $t\in [1,2]$ s were exported for analysis from simulations in P1-IN, P1-OUT, P2-IN, and P2-OUT.
